# *In Vitro* and *In Vivo* Differences in Murine Third Complement Component (C3) Opsonization and Macrophage/Leukocyte Responses to Antibody-Functionalized Iron Oxide Nanoworms

**DOI:** 10.3389/fimmu.2017.00151

**Published:** 2017-02-15

**Authors:** Guankui Wang, James I. Griffin, Swetha Inturi, Barbara Brenneman, Nirmal K. Banda, V. Michael Holers, Seyed Moein Moghimi, Dmitri Simberg

**Affiliations:** ^1^The Skaggs School of Pharmacy and Pharmaceutical Sciences, University of Colorado Denver, Anschutz Medical Campus, Aurora, CO, USA; ^2^Division of Rheumatology, School of Medicine, University of Colorado Denver, Anschutz Medical Campus, Aurora, CO, USA; ^3^School of Medicine, Pharmacy and Health, Durham University, Queen’s Campus, Stockton-on-Tees, UK

**Keywords:** iron oxide, leukocyte, complement, antibody, PEG

## Abstract

Balancing surface functionalization and low immune recognition of nanomedicines is a major challenge. Opsonization with the third component of the complement protein (C3) plays a major role in immune cell recognition of nanomedicines. We used dextran-coated superparamagnetic iron oxide nanoworms (SPIO NWs) to study the effect of surface functionalization on C3 opsonization in mouse serum and subsequent macrophage/leukocyte recognition *in vitro* as well as on intravenous injection into mice. Previously, we found that in mouse serum, SPIO NWs became opsonized with C3 *via* complement lectin pathway. Crosslinking the dextran shell with epichlorohydrin significantly decreased C3 opsonization and uptake by mouse peritoneal macrophages. Crosslinked nanoworms (NWs) further functionalized with polyethylene glycol (PEG) or with PEG-antibody (Ab) (~160 IgG molecules/particle) did not show an increase in C3 opsonization and peritoneal macrophage uptake *in vitro*. Following tail vein injection into mice, plain crosslinked NWs and PEGylated crosslinked NWs showed very low C3 opsonization and mouse leukocyte uptake. However, Ab-decorated crosslinked NWs showed significant C3 opsonization and high level of complement-dependent uptake by leukocytes in mice. Decreasing the number of conjugated Abs to 46 IgG molecules/particle significantly reduced C3 opsonization and leukocyte uptake. Using fresh mouse lepirudin plasma rather than serum showed better correlation with C3 opsonization *in vivo*. The reason for this difference could be related to the known instability of complement classical pathway in mouse sera. Our data illustrate that fine-tuning in nanoparticle surface functionalization with Abs is required to avoid excessive complement activation and complement-mediated immune uptake in mice, and raise issues with *in vitro* immunological assays of nanomedicines intended to mimic *in vivo* conditions.

## Introduction

The success of systemically and locally administered nanomaterials largely depends on the ability of nanosized carriers to efficiently evade the immune system (1). Several pathways of the innate immune system mediate clearance of nanoparticles by phagocytic cells. Complement system is an effector arm of the innate immune system composed of more than 30 blood proteins that accounts for about 5% of globulins in serum and is responsible for recognizing, eliminating, and destroying pathogens (2). The activation of complement system on foreign surface *via* lectin, classical, or alternative pathways [LP, classical pathway (CP), or AP, respectively] converges to cleave native C3 and generate a highly reactive thioester on C3b, which covalently attaches to reactive functional groups (e.g., hydroxyl and amines) on target surface (3–5). Opsonization by C3b and its cleavage products (iC3b, C3d) triggers particle recognition by leukocytes through complement receptors (6, 7), whereas soluble cleavage byproducts C3a and C5a are among the most potent anaphylatoxins and proinflammatory molecules with low nanomolar affinity (8). Numerous nanomaterials activate the complement system and become opsonized with C3 *in vitro* and *in vivo* (9–13).

**Figure 1:**
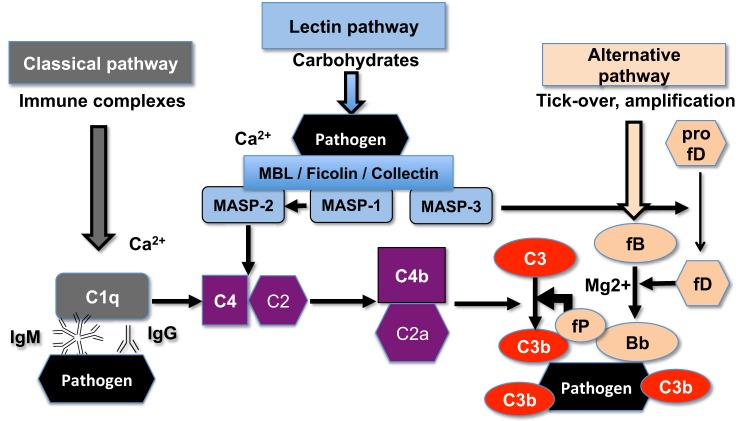
**The scheme of the upstream mouse complement pathways**. C5a convertase and the terminal membrane attack complex pathway are not shown. There are three initiating complement pathways: classical, lectin, and alternative. All the pathways generate activated central complement protein component C3, liberating C3b, and its fixation on the pathogen (or nanoparticle) surface.

Superparamagnetic iron oxide (SPIO) nanoparticles have been used as magnetic resonance imaging (MRI) contrast agents and also as carriers for drug delivery (14). SPIO nanoparticles consist of 5–8 nm magnetite–maghemite (Fe_3_O_4_ and γ-Fe_2_O_3_) crystalline cores coated with a polymer (15). Recently, we reported the synthesis of 20 kDa dextran-coated superparamagnetic iron oxide nanoworms (SPIO NWs) with high transverse relaxivity r2, which makes them promising MRI contrast agents (16). Unfortunately, SPIO NWs potently activate complement in both mice and humans (17). Previously, we demonstrated that mouse complement activation is *via* LP (17), whereas human complement activation is predominantly *via* the AP (18). As shown in Figure 1, initiation of the LP starts with the binding of mannose-binding lectin (MBL)-A/C, ficolins, or collectin-11 to the carbohydrates on the pathogen surfaces. The binding leads to activation of MBL-associated serine proteases (MASPs), leading to formation of the complement convertase C4bC2a, cleavage of C3, deposition of initial C3b, and possible amplification *via* the alternative pathway convertase C3bBb. MASP-2 plays a direct role in formation of the complement convertase C4bC2a (19) whereas MASP-1 and MASP-2 indirectly activate MASP-2 (20) and factor D (21, 22), respectively.

Interestingly, modifying the surface dextran coating with epichlorohydrin (ECH) [resulting in poly-(2-hydroxypropyl ether) hydrogel] blocked mouse complement C3 opsonization and leukocyte uptake (16). At the same time, it is not clear how further surface functionalization of ECH-crosslinked NWs (hereafter CL-NWs) affects complement activation and immune uptake. In view of the remarkable redundancy of pathways responsible for immune recognition (7) maintaining the delicate balance between surface functionalization and stealth properties could be a challenging task. In particular, the effect of addition of targeting ligands on “stealth” nanoparticles on complement activation and immune uptake has not been investigated in depth. In order to understand the impact of surface functionalization of CL-NWs with targeting antibodies (Abs) on complement activation and immune recognition, we modified CL-NWs with a polyethylene glycol (PEG) linker followed by a model anticancer Ab (trastuzumab). Here, we demonstrate that surface modification of CL-NWs that have low complement activation with trastuzumab can increase complement activation dependent on Ab surface density, and further raise an issue of *in vitro* versus *in vivo* correlation assays of immune recognition of nanoparticles.

## Results and Discussion

### Effect of Nanoworm (NW) Modification and Functionalization on C3 Opsonization and Immune Uptake in Mouse Sera *In Vitro*

**Figure 2:**
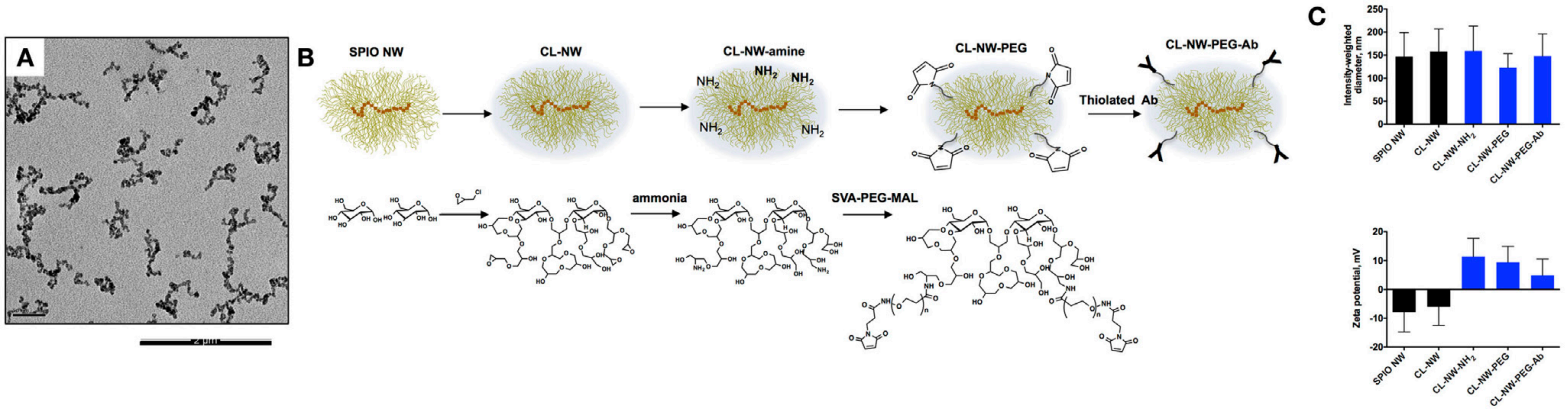
**Surface modification of superparamagnetic iron oxide nanoworms (SPIO NWs)**. **(A)** Representative transmission electron microscopy (TEM) image of SPIO NWs shows electron dense worm-like aggregates of magnetite crystals. Only the cores are visible in TEM. The scale bar is 200 nm; **(B)** graphic (top) and chemical (bottom) description of modifications of SPIO NWs. Dextran layer (yellow) was crosslinked with epichlorohydrin to form crosslinked hydrogel nanoworms (CL-NWs). The CL-NWs were then aminated using different concentrations of ammonium hydroxide to form CL-NWs-amine. CL-NWs-amine were further modified with succinimidyl valerate (SVA)-polyethylene glycol (PEG) maleimide and conjugated to thiolated antibodies (Abs); **(C)** the upper graph shows hydrodynamic size of nanoworms (NWs) measured by dynamic light scattering. The surface modifications did not significantly affect the hydrodynamic size of NWs. The lower graph represents the zeta potential values of NWs. The overall electric charge of CL-NWs switched from negative to positive after amination and for the corresponding functionalized particles.

Dextran-coated SPIO NWs were prepared from 20 kDa dextran, FeCl_2_, and FeCl_3_ by a modified Molday and MacKenzie precipitation protocol (23). According to transmitted electron microscopy (Figure 2A), nanoparticle cores contained worm-like aggregates of iron oxide crystals. On the outside, the cores are covered with a shell of dextran chains (Figure 2B). Dextran shell was crosslinked with ECH in the presence of NaOH to yield hydrogel-coated CL-NWs (Figure 2B). The residual epoxides on CL-NWs were treated with ammonia to generate primary amines that were further functionalized with heterobifunctional maleimide (MAL)-PEG_3400_-succinimidyl valerate (SVA) to yield CL-NWs-PEG-MAL (hereafter CL-NWs-PEG; Figure 2B). PEG-MAL-functionalized particles were modified with thiolated Ab trastuzumab (Herceptin^®^) to yield CL-NWs-PEG-Ab (Figure 2B). According to size measurements (Figure 2C, top), the modifications did not affect the hydrodynamic diameter of NWs. Zeta potential values were slightly negative for SPIO NWs and CL-NWs, but expectably became positive for CL-NWs treated with ammonia and the corresponding functionalized particles (Figure 2C, bottom). Quantitative measurements (see Materials and Methods) showed that on average there were ~160 IgG molecules per each CL-NWs-PEG-Ab nanoparticle.

**Figure 3:**
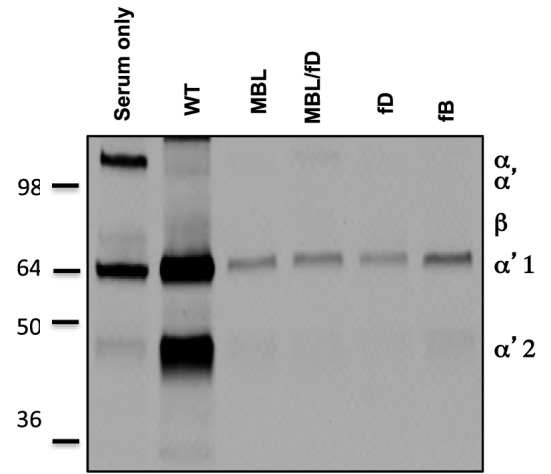
**Mechanisms of complement C3 opsonization by non-modified superparamagnetic iron oxide nanoworms (SPIO NWs)**. Detection of C3 (mostly in iC3b form due to appearance of α2′ fragment at ~40 kDa) bound to SPIO NWs in mouse sera. Nanoworms became opsonized in control (wild type) sera but not in sera deficient for mannose-binding lectin (MBL), MBL/factor D, factor D, and factor B, suggesting that the LP is the initiating route and the AP turnover is responsible for amplification. Note that beta-chain of mouse C3 is not highly detectable with this antibody.

In order to confirm the role of the LP as the inciting pathway of complement activation in mouse serum, SPIO NWs were incubated in validated sera obtained from mice deficient for various complement pathways (Figure 1), washed, and analyzed for mouse C3 in Western blotting (the same amount of particles were used in the assay and loaded on the gel). According to Figure 3, SPIO NWs showed strong C3 (C3b/iC3b) opsonization in wild type (WT) mouse serum, whereas the opsonization was blocked in sera deficient for MBL-A/C (LP) and MBL-A/C/factor D (LP and AP). In addition, C3 opsonization was blocked in sera deficient for factor D and factor B (AP). These data confirm that complement is triggered by MBL/MASP-2-dependent LP activation, whereas the AP provides the amplification loop.

**Figure 4:**
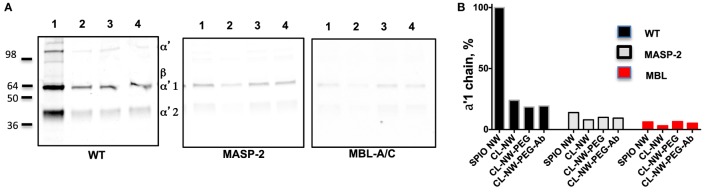
**Crosslinked dextran blocks lectin pathway activation in mouse sera**. **(A)** Detection of C3 bound to superparamagnetic iron oxide nanoworms (SPIO NWs) and CL-NWs in a wild type (WT) mouse serum and in serum deficient for lectin pathway factors MBL-associated serine protease (MASP)-2 and mannose-binding lectin (MBL)-A/C. The lanes represent: 1, SPIO NWs; 2, CL-NWs; 3, CL-NWs-polyethylene glycol (PEG); and 4, CL-NWs-PEG-Ab (160 IgG/particle). Left gel = WT serum. Complement opsonization was decreased for all CL-NWs formulations. C3 opsonization was blocked for all groups of nanoworms; **(B)** densitometry analysis of C3 α′1-chain. All CL-NWs formulations showed >80% decrease of C3 binding in WT sera compared to SPIO NWs. The residual complement activation of CL-NWs and functionalized CL-NWs is *via* the lectin pathway.

Next, we compared the efficiency of C3 opsonization of CL-NWs, CL-NWs-PEG, and CL-NWs-PEG-Ab with SPIO NWs. Particles were incubated in normal mouse serum or in sera deficient for the LP factors MBL-A/C and MASP-2, washed, loaded in the same amount on a gel, and analyzed by Western blotting. All CL-NWs formulations showed 80–85% decrease in C3 opsonization in normal serum compared with SPIO NWs (Figures 4A,B). Moreover, CL-NWs formulations incubated in MBL-A/C-deficient and MASP-2-deficient sera (Figures 4A,B) showed further reduction of C3 opsonization. These data demonstrate that crosslinking predominantly blocks complement activation and functionalization of CL-NWs with PEG or PEG-Ab does not enhance complement activation in mouse serum.

**Figure 5:**
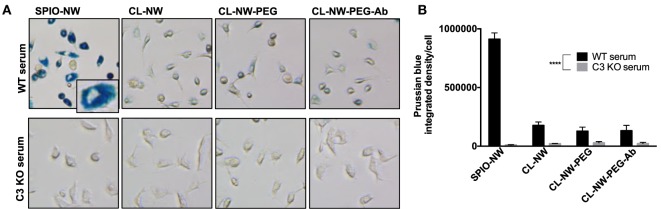
**Peritoneal macrophage uptake of superparamagnetic iron oxide nanoworms (SPIO NWs) and CL-NWs in mouse serum is complement dependent**. **(A)** Prussian blue staining of iron uptake by cells. The peritoneal macrophages were incubated with SPIO NWs, CL-NWs, CL-NWs-polyethylene glycol (PEG), and CL-NWs-PEG-Ab (from left to right) in the presence of wild type (WT) serum (first row) or C3-deficient [knockout (KO)] serum (second row). All images were taken at 20× magnification and cropped to the same extent. Most of the Prussian blue stain is clearly intracellular as shown in the magnified insert; **(B)** quantification of Prussian blue staining of iron in cells. The uptake of CL-NWs was 85% decreased compared with SPIO NWs (*n* = 20; *p* < 0.0001). The uptake of nanoworms C3 KO serum was significantly less than in WT sera (*n* = 20; *p* < 0.0001). For quantification and statistical analysis, see Section “Materials and Methods.”

C3 is the critical opsonin mediating the uptake of foreign pathogens by macrophages and leukocytes (2). We tested whether functionalization of CL-NWs affected the uptake by non-activated mouse peritoneal macrophages in mouse serum. As shown in Figure S1 in Supplementary Material, over 70% of freshly isolated cells showed expression of CD11b (receptor for iC3b) and F4/80 (a macrophage marker). SPIO NWs, CL-NWs, CL-NWs-PEG, and CL-NWs-PEG-Ab were preincubated in normal (WT) mouse serum for 15 min, and then added to the cells at 0.1 mg/mL Fe for 6 h. According to Prussian blue staining and quantification (Figures 5A,B, see Materials and Methods for details), SPIO NWs showed highly intense cytoplasmic accumulation of iron. CL-NWs showed 80% less uptake than SPIO NWs. The functionalized CL-NWs had the same level of residual uptake as non-functionalized CL-NWs, suggesting that PEG and Ab modifications do not trigger the uptake in mouse serum. Incubation of SPIO NWs and all CL-NWs formulations in C3-deficient mouse serum resulted in a complete blockade of the residual uptake of CL-NWs formulations (Figures 5A,B), suggesting that the uptake of SPIO NWs and the residual uptake of CL-NW formulations are mediated through C3 opsonization.

### Effect of Cl-NWs Functionalization on C3 Opsonization and Immune Uptake *In Vivo*

**Figure 6:**
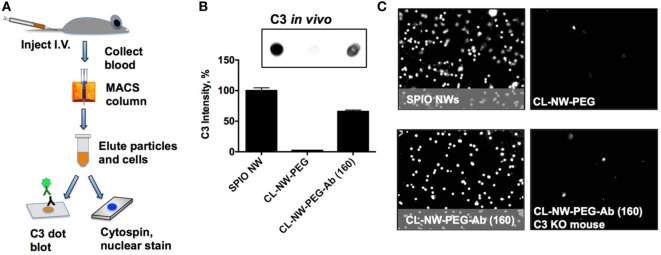
**Conjugated antibodies trigger complement activation and leukocyte uptake *in vivo***. **(A)** Nanoworms (NWs) were injected intravenously into mice and recovered from the blood 5 min post-injection. MIDI Max magnetic column trapped both free NWs and magnetically labeled leukocytes that took up NWs; **(B)** NWs were loaded on nitrocellulose membrane (same amount of Fe for all formulations) and analyzed for mouse C3. The image shows the C3 dot blot and the graph shows quantitative image analysis. There was no complement opsonization of CL-NWs-polyethylene glycol (PEG) but significant opsonization of CL-NWs-PEG-Ab (160 IgG/particle); **(C)** microscopic images of slides after cytospin (nuclear Hoechst stain shows nucleated leukocytes) that were eluted from magnetic column. One representative cropped microscopic field is shown for each formulation. Each dot represents a leukocyte. A significant number of leukocytes took up particles after injection of superparamagnetic iron oxide nanoworms (SPIO NWs) and CL-NWs-PEGs-Ab. The uptake by leukocytes was abolished when CL-NWs-PEG-Ab were injected into C3-deficient mouse (lower right).

Previously, we demonstrated that complement C3 mediates the uptake of SPIO NWs by blood leukocytes in mice (10). SPIO NWs, CL-NWs-PEG, and CL-NWs-PEG-Ab were injected intravenously into mice at 5 mg Fe per kg body weight, recovered from blood 5 min post-injection using a magnetic column [in this process, both free particles and magnetically labeled leukocytes were enriched (10)], and analyzed for C3 opsonization and leukocyte uptake as shown in Figure 6A. We used a previously established (10, 16–18) dot-blot procedure to compare the levels of C3 opsonization on NWs. According to Figure S2 in Supplementary Material, C3 dot-blot assay correlates with C3 Western blot for determining the levels of C3 on particles. According to Figures 6B,C, SPIO NWs showed high level of C3 opsonization *in vivo* and leukocyte uptake. Similar to *in vitro* serum results, CL-NWs-PEG showed less than 10% of C3 compared to SPIO NWs, and low level of leukocyte uptake. Non-modified CL-NWs also showed low level of *in vivo* C3 opsonization and leukocyte uptake (Figure S3 in Supplementary Material), confirming our previous findings (10). However, in contrast to *in vitro* measurements, CL-NWs-PEG-Ab decorated with ~160 IgG/particle showed 66% of C3 opsonization of SPIO NWs and high leukocyte uptake *in vivo* (Figures 6B,C). Injection of CL-NWs-PEG-Ab into C3 knockout (KO) mice that completely lacks C3 abolished the leukocyte uptake (Figure 6C). These data suggest that conjugation of IgG on the particles triggered complement activation and complement-dependent immune uptake that was not detected using *in vitro* assays in mouse serum.

**Figure 7:**
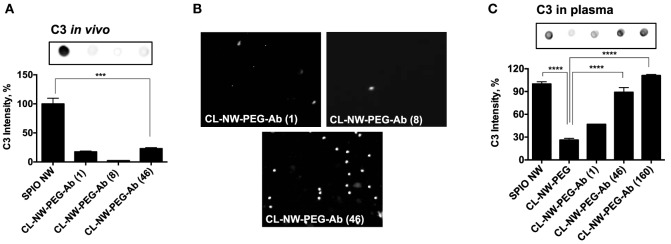
**Decreasing complement activation and leukocyte uptake *in vivo* by controlling number of antibodies per particle**. **(A)** Complement opsonization *in vivo* of CL-NWs prepared with reduced number of IgG molecules per particle. Superparamagnetic iron oxide nanoworms (SPIO NWs) were used as a reference (100%) of C3 levels. Complement opsonization was significantly decreased for CL-NWs-polyethylene glycol (PEG)-Ab with 1 IgG/particle, 8 IgG/particle, and 46 IgG/particle compared to SPIO NWs (*n* = 3; *p* < 0.0001); **(B)** leukocyte uptake *in vivo* shows that CL-NWs-PEG-Ab with low number of IgG per particle show much lower leukocyte uptake than CL-NWs-PEG-Ab (160 IgG/particle, compared to Figure 6C); **(C)** complement C3 opsonization in fresh plasma *in vitro*. Particles were incubated in fresh plasma obtained with lepirudin as anticoagulant. C3 opsonization for CL-NWs-PEG-Ab was much higher in plasma than in serum (Figure 3) but also much higher than *in vivo*, suggesting that *in vitro* opsonization assay in plasma, while more representative than serum, still does not fully correlate with *in vivo* opsonization (*p* = 0.0001 for 46 IgG/particle and 160 Ab/particle, respectively).

Surface immobilized Abs and immune complexes, however, are known to trigger complement *via* the CP (24). It is likely that *in vivo* Ab-functionalized NWs trigger the CP. At least two surface-bound IgG molecules must be bridged by a C1q molecule before activation of the CP can proceed. In order to test whether the number of IgG per particle can control complement activation *in vivo*, we synthesized CL-NWs-PEG-Ab bearing different Ab densities and tested their C3 opsonization and leukocyte uptake *in vivo*. According to Figures 7A,B, CL-NWs-PEG-Ab with 1 IgG/particle, 8 IgG/particle, and 46 IgG/particle showed significantly lower levels of *in vivo* C3 opsonization (17, 3, and 22% of SPIO NWs, respectively) and leukocyte uptake than CL-NWs-PEG-Ab with 160 IgG/particle. However, the observation of higher C3 opsonization with CL-NW-PEG-Ab bearing a single Ab molecule compared with CL-NW-PEG-Ab with 8 IgG/particle is intriguing. The reason for this is unclear, but this suggests the involvement of other *in vivo* factors regulating complement activation and fixation and therefore requires further investigation. Nevertheless, these experiments suggest that decreasing surface density of Ab molecules can suppress complement activation and immune cell recognition *in vivo*. CL-NWs-PEG-Ab with 8 trastuzumab/particle showed specific uptake by HER2/neu + human breast cancer cell line SKBR-3 (Figure S4 in Supplementary Material), suggesting that the immobilized Ab is functional on the nanoparticles and therefore may bind to its designated target *in vivo*.

Next, we sought an explanation to the observed lack of complement activation by CL-NWs-PEG-Ab (160 IgG/particle) in serum (Figure 4) versus *in vivo*. Previous evidence suggested that the CP of the complement system is unstable in mouse sera (25–27) and, furthermore, starts losing its activity at room temperature (RT) and even during −70°C storage (26). Accordingly, blood clotting procedures and serum isolation steps could trigger loss of CP activity and explain poor C3 opsonization in mouse serum through this pathway. To address this, we repeated the experiments with fresh mouse plasma using recombinant hirudin (lepirudin) as anticoagulant. Lepirudin is the only known anticoagulant that does not interfere with complement activation. The results shown in Figure 7C demonstrate that SPIO NWs potently activate complement in fresh lepirudin plasma. CL-NWs-PEG showed only 26% of C3 opsonization of SPIO NWs in plasma. At the same time, CL-NWs-PEG-Ab (1 IgG/particle), CL-NWs-PEG-Ab (46 IgG/particle), and CL-NWs-PEG-Ab (160 IgG/particle) showed high C3 opsonization compared to SPIO NWs [46, 88, and 110% of C3 opsonization to SPIO NWs, respectively (Figure 7C)]. Therefore, the results in plasma are better correlated with C3 opsonization *in vivo* than in serum. At the same time, the relative levels of C3 opsonization were much higher in plasma than *in vivo*. Thus, plasma C3 opsonization of CL-NWs-PEG-Ab (46 IgG/particle) was 88% of SPIO NWs (Figure 7C), whereas *in vivo* C3 opsonization level of the same particle was 22% of SPIO NWs (Figure 7A). These discrepancies may be related to the dynamic differences in NW protein corona *in vitro* versus *in vivo* conditions in regulating complement activation (28). In summary, these data suggest that C3 assay in plasma is a better predictor of *in vivo* complement activation for Ab-modified particles than in serum, but at the same time the opsonization efficiency *in vitro* does not fully correlate with the efficiency *in vivo*, likely due to differences in dynamics of protein interaction, corona formation, and complement activation.

Our studies demonstrate that surface modifications of nanoparticles have profound effect on complement C3 opsonization and the resultant immune uptake. Previous studies using PEGylated liposomes and nanoparticles showed loss of stealth properties after tethering of Abs and ligands (29–31) and that surface functionalization affects the cellular uptake (32–34). Nevertheless, the mechanistic studies explaining the effect of surface functionalizations were lacking and our study demonstrates for the first time the balance between surface functionalization, complement activation, and immune cell uptake. Immune system, including the complement system, is generally redundant, meaning that multiple pathways are utilized to recognize foreign epitopes. While the exact pathway by which surface Ab triggers complement activation needs to be elucidated further, the need to control complement activation *via* the number of conjugated Abs is an important aspect in the design of targetable nanomedicines.

Importantly, our study suggests that complement activation *via* surface immobilized Ab could be missed using *in vitro* assays in murine sera. Therefore, valid complement assays of nanoparticles should be used, at the very least using lepirudin plasma *in vitro*, but preferably in the animal *in vivo*. The lack of correlation between *in vitro* and *in vivo* assays adds to the lack of correlation in pathways of complement activation in mice and humans (10, 17). Mouse models are widely used in preclinical studies of biodistribution and toxicity of drug delivery systems. In addition, complement-mediated mechanisms of immune uptake are likely similar in mice and humans (10). Despite these, any extrapolation of mouse data on the immune recognition of nanocarriers to humans should be interpreted with caution.

## Materials and Methods

### Materials

All reagents used for NW synthesis including Fe salts and 20 kDa (range 15–25 kDa) dextran were purchased from Sigma-Aldrich (St. Louis, MO, USA). Cell culture media were purchased from Corning Life Sciences (Corning, NY, USA). Copper grids (300 mesh) were purchased from Electron Microscopy Sciences (Hatfield, PA, USA). Anti-HER2 Ab Herceptin^®^ was generously donated by the Pharmacy of the University of Colorado Cancer Center, Anschutz Medical Campus. Anti-mouse anti-C3 Ab was purchased from MP Biomedicals (Solon, OH, USA). IRDye 800CW-labeled secondary Abs were purchased from Li-COR Biosciences (Lincoln, NE, USA). Purified anti-mouse/human CD11b Ab was purchased from BioLegend (San Diego, CA, USA). Anti-mouse F4/80 Ab was purchased from Caltag Medsystems Ltd. (Buckingham, UK). Hoechst 33342 for nuclei staining was purchased from Thermo Fisher Scientific (Waltham, MA, USA). The Abs for each experiment were diluted according to the recommendations from manufacturers. Mouse sera deficient for C3, MBL-A/C, MBL-A/C/factor D, and factor B were collected from mice that were bred in an animal vivarium at the University of Colorado Anschutz Medical Campus according to the Institutional Animal Care and Use Committee (IACUC) approved breeding protocol. Recombinant human hirudin (lepirudin, catalog No. ACM154) was obtained from Aniara Diagnostica, LLC (West Chester, OH, USA), reconstituted in water to 1 μg/μL (160 antithrombin units/μL), and stored aliquoted at −80°C. Lepirudin anticoagulated plasma (hereafter plasma) was obtained by collecting blood through the cardiac puncture (final lepirudin concentration 3–4 μg/mL) and centrifuging the tube at 2,000 *g* for 15 min.

### Synthesis of SPIO and Crosslinked Nanoworms (CL-NWs)

Nanoworms were synthesized by a modified one-pot Molday and MacKenzie (23) precipitation method as described earlier (16). Nanopure water (30 mL) was de-oxygenated with nitrogen gas and used to dissolve 6 g dextran (molecular weight 20 kDa, Sigma-Aldrich), 1.26 g Fe(III) chloride, and 0.498 g Fe(II) chloride in a round bottom flask. Next, 2.4 mL of cold 25% (v/v) ammonium hydroxide (NH_4_OH) was slowly added to the mixture of dextran and iron salts under nitrogen atmosphere with rapid stirring on ice. After formation of NWs, the mixture was heated at 80°C with stirring. After cooling, SPIO NWs were purified overnight using a 20 kDa dialysis cassette (Thermo Fisher Scientific, Waltham, MA, USA) against double distilled water to remove free dextran. The particles were chemically crosslinked using 1-chloro-2, 3-epoxypropane (ECH) with sodium hydroxide as described before (16). SPIO NWs and CL-NWs were filtered through a 0.45 μLm-pore filter (Millipore, Billerica, MA, USA) prior to use.

### Surface Modifications of CL-NWs

CL-NWs were aminated by adding different concentrations of ammonium hydroxide at 4°C overnight to form CL-NWs-NH_2_ and dialyzed for 24 h to remove free NH_4_OH. For the Ab conjugation, MAL-PEG-SVA (MAL-PEG-SVA, Laysan Bio) was reacted at excess with CL-NWs-NH_2_ at RT for 30 min to form CL-NWs-PEG-MAL or CL-NWs-PEG. Anti-HER2 Ab Herceptin^®^ was reacted in the last step with MAL-PEG to form CL-NWs-PEG-Ab. NWs were purified by ultracentrifugation at 55,000 rpm, filtered through a 0.45 μm pore filter, and finally, stored in Dulbecco’s phosphate-buffered saline (DPBS) at pH 7.4 before use.

### Characterization of NWs

Transmission electron microscopy imaging was conducted to visualize the NWs using FEI Tecnai Spirit BioTwin electron microscope (Electron Microscopy Facility at the University of Colorado Boulder) at a 100 kV working voltage. Size measurements were done in DPBS and zeta potential measurements were done in 0.1× DPBS at RT using a Zetasizer Nano ZS (Malvern Instruments Ltd., Malvern, UK). The intensity-weighted size distribution peak value was used to report hydrodynamic diameters of the NWs. To quantify the Ab on the NWs, known amount of CL-NWs-PEG-Ab (0.2 μg Fe) was applied in triplicates onto a 0.2 μm pore nitrocellulose membrane (Bio-Rad). Standard dilutions of free trastuzumab were also applied onto the membrane to generate a standard curve. The membrane was blocked with 5% (w/w) non-fat dry milk in PBS-T (DPBS with 0.1% Tween^®^ 20) for 1 h at RT and probed with IRDye 800CW-labeled antihuman Ab. The membrane was scanned with Odyssey infrared imager (Li-COR Biosciences, Lincoln, NE, USA). The integrated dot intensity in the scanned images was determined from 16-bit grayscale images using ImageJ software and plotted using Prism 6 software (GraphPad Software, Inc., La Jolla, CA, USA) to determine the number of Ab molecules per spot using the calibration curve. Concentration of particles per milligram Fe was determined with NanoSight (Malvern Instruments) and Fe concentration was determined with ferrozine iron assay as described before (16). The number of Abs per NW was determined after dividing the number of Abs per spot by the number of NWs per spot.

### Protein Binding Assay

Superparamagnetic iron oxide and CL-NWs (10 μL of 1 mg/mL) were incubated with 30 μL of mouse serum or lepirudin plasma for 15 min at RT. At the end of incubation, particles were washed three times with 1× PBS by centrifugation at 100,000 *g* at 4°C using Beckman Optima TLX ultracentrifuge. The pellets were resuspended in 20 μL DPBS, and the concentration of Fe in each sample was normalized with ferrozine iron assay as described before (16). For complement C3 western blot, 10 μL aliquots were used for gel electrophoresis. The samples were mixed with loading buffer [denaturing buffer containing 100 mM Tris, 20% glycerol, 4% SDS, 5% (v/v) 2-mercaptoethanol, 0.02% bromophenol blue] and then boiled at 95°C for 5 min. After cooling for 5 min, the samples and marker proteins (Precision Plus Proteins Dual Color Standards from Bio-Rad) were loaded onto Mini-PROTEAN TGX Gels (Bio-Rad, Hercules, CA, USA) and separated at 50 V for 5 min and then 100 V for 90 min. Gels were then transferred to nitrocellulose membranes using the Mini Trans-Blot cell system overnight at 50 V at 4°C. For C3 dot blot, 2 μL aliquots were applied in triplicates onto a nitrocellulose membrane. The membranes were blocked using 5% non-fat dry milk in DPBS-T (DPBS with 0.1% Tween^®^ 20) at RT for 1 h, probed with corresponding primary Abs at RT for 1 h, followed by washing the membranes 3× with DPBS-T, and finally, 1 h incubation with the corresponding IRDye 800CW-labeled secondary Abs against the primary Ab species (see Materials). The membranes after immunoblotting were visualized using an Odyssey infrared imager. The integrated dot intensity in the scanned images was quantitatively analyzed using ImageJ software and plotted with Prism 6 software as described above.

### Uptake of NWs *In Vitro*, Prussian Blue Staining, and Quantification

Human breast cancer cell line SKBR-3 cells were maintained in McCoy’s 5A medium (ATCC) supplemented with 10% fetal bovine serum. Mouse peritoneal macrophages were obtained by peritoneal lavage with 5 mL ice cold PBS, post-mortem. For experiment, cells were plated into 96-well plate. For uptake experiments, NWs were preincubated for 15 m with WT mouse sera or C3 KO sera and added at 0.1 mg/mL Fe concentration to cells for 6 h. After the incubation, cells were washed using DPBS for three times, fixed in 4% paraformaldehyde at 4°C overnight, and then stained using Prussian blue for 1 h. Prussian blue staining is the standard method for detecting iron in cells and tissues, and has been extensively used for detecting uptake of iron oxide nanoparticles by our group and others (35, 36). The method is based on formation of insoluble, blue colored coordination complexes between Fe^3+^ and potassium ferrocyanide. Maghemite crystals of SPIO contain both Fe^3+^ and Fe^2+^, and the surface of crystals is always oxidized to Fe^3+^, so the complete degradation of nanoparticles is not required for the staining to work. In order to quantify the blue color of the complexes inside the cells, TIFF RGB images of stained cells were acquired with a Nikon Eclipse E600 microscope using same exposure and magnification. The images were combined into a gallery with Adobe Photoshop and color balance was adjusted with a Level tool to make the cell-free area white. The gallery image was exported into ImageJ, converted into YUV color space, and thresholded for blue and green components using a Threshold Color plugin. Then, the image was converted into a 16-bit gray scale and inverted. The background was completely subtracted with a Math Subtraction tool. A ROI was drawn around each cell and integrated pixel density was measured. The data were plotted as means and SD using Prism software. An average of at least 20 cells was used.

### Nanoparticle Uptake *In Vivo*

Wild type and *C3^−/−^* mice (Jax Laboratories: B6.129S4-*C3^tm1Crr/^*J) were bred in house according to the approval by University of Colorado Animal Protocol Committee. NWs were injected as a 5 mg/kg bolus *via* tail vein into WT and C3 KO mice (8 weeks of age, females). Following the injection (5 min), mice were sacrificed and the blood was drawn *via* cardiac puncture using heparin as anticoagulant. Blood was applied on Miltenyi Mini MACS magnetic column (Miltenyi Biotech) and the trapped cells and particles were washed extensively with PBS. The particles and magnetic leukocytes were then eluted from the column. The cells were pelleted with tabletop Eppendorf centrifuge and the particles in the supernatant were further concentrated with ultracentrifuge for C3 dot-blot assay. The cell pellet was resuspended in 200 μL PBS and the cells were concentrated on slides using Shandon Cytospin 4 centrifuge (Thermo Fischer), fixed with 10% (v/v) formalin in PBS, and stained with Hoechst dye (Thermo Fischer) to enable leukocyte nuclei visualization. The images were taken with Nikon Eclipse E600 fluorescent microscope using DAPI filter at low (40×) magnification.

### Statistical Analysis

The statistical analysis was performed using Prism 6 software. The differences between means of experimental groups were analyzed using a two-tailed parametric *t*-test assuming 95% confidence interval. Data shown as means ± SD. Differences in all data are shown as *p < 0.05; ***p < 0.001; ****p < 0.0001.

## Ethics Statement

This study was carried out in accordance with the guidelines of the University of Colorado Office of Animal Care. The protocol was approved by the University of Colorado IACUC.

## Author Contributions

GW designed and performed experiments, and analyzed data. JG performed experiments. SI performed experiments. BB performed experiments. NB analyzed data and provided reagents. VH analyzed data and provided reagents. SM designed experiments, analyzed data, and edited the paper. DS designed experiments, analyzed data, and wrote the paper.

## Conflict of Interest Statement

The authors declare that the research was conducted in the absence of any commercial or financial relationships that could be construed as a potential conflict of interest. The reviewer YL declared a shared affiliation, though no other collaboration, with several of the authors to the handling Editor, who ensured that the process nevertheless met the standards of a fair and objective review.
