# Supplementary material for: In Vitro and In Vivo Differences in Murine Third Complement Component (C3) Opsonization and Macrophage/Leukocyte Responses to Antibody-Functionalized Iron Oxide Nanoworms
Source: Front Immunol. 2017 Feb 15;8:151. doi: 10.3389/fimmu.2017.00151 (PMC5309246; doi:10.3389/fimmu.2017.00151)
Supplement: Supplementary file 1 [file Data_Sheet_1.PDF]

Supplemental data:

In vitro and in vivo differences in murine third complement component (C3) opsonization and macrophage/leukocyte responses to antibody-functionalized iron oxide nanoworms.

Guankui Wang[1], James I Griffin[1], Swetha Inturi[1], Barbara Brenneman[1], Nirmal K. Banda[2], V. Michael Holers[2], Seyed Moein Moghimi[3] and Dmitri Simberg[1,*]

[1]The Skaggs School of Pharmacy and Pharmaceutical Sciences, University of Colorado Denver, Anschutz Medical Campus, 12850 E. Montview Blvd., Aurora, CO 80045, USA

[2]Division of Rheumatology, School of Medicine, University of Colorado Denver, Anschutz Medical Campus, 1775 Aurora Court, Aurora, CO 80045, USA

[3]School of Medicine, Pharmacy and Health, Durham University, Queen's Campus, Stockton-on-Tees TS17 6BH, UK

.

[*] *Corresponding author: Dmitri.Simberg@ucdenver.edu*

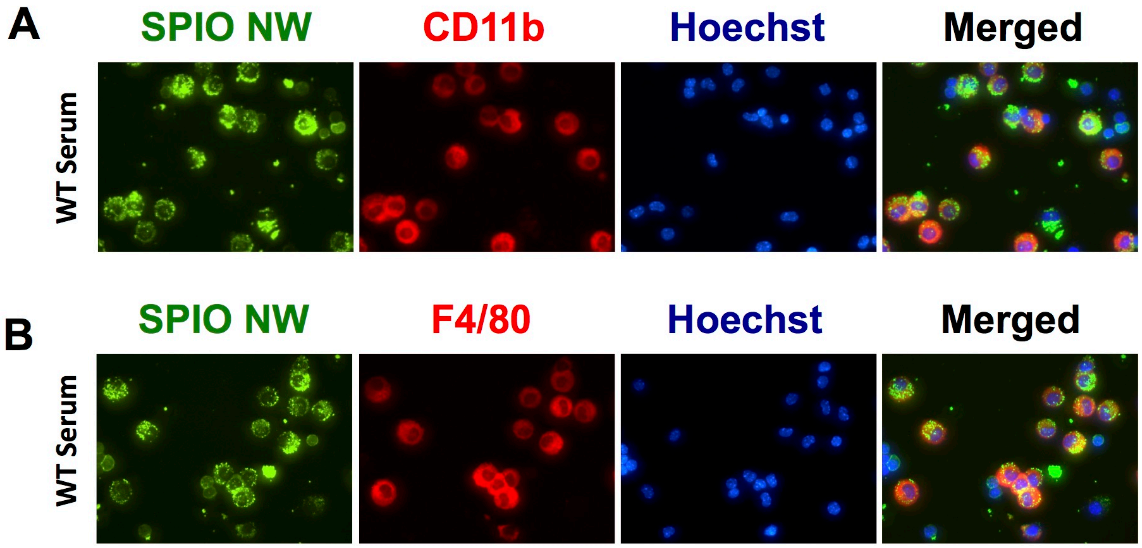

Supplemental Fig. S1: Freshly isolated peritoneal macrophages. Cells were stained for CD11b (Mac-1 receptor for complement) or F4/80 (macrophage marker) and for dextran (green-nanoparticles). The cells were over 70% positive for the markers and the staining colocalized with nanoparticles.

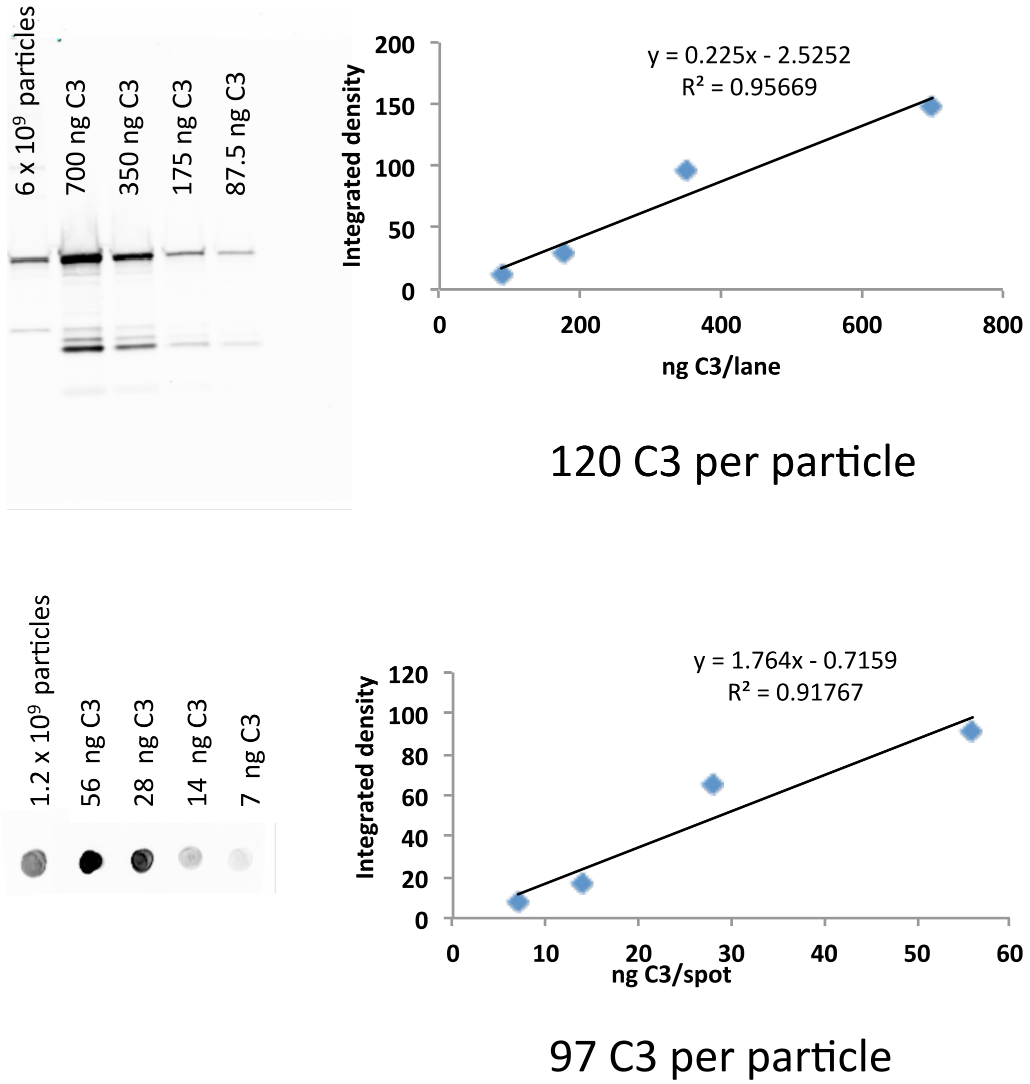

Supplemental Fig. S2: Comparison between C3 western blot (upper gel and graph) and C3 dot blot (lower gel and graph). SPIO NWs were incubated in serum, washed and loaded on a gel or a membrane together with the dilutions of purified C3 (standard curve). The graph shows the standard curve of band/dot integrated densities measured by ImageJ. When calculated per nanoparticle, the number of C3 molecules was similar for WB and DB assays.

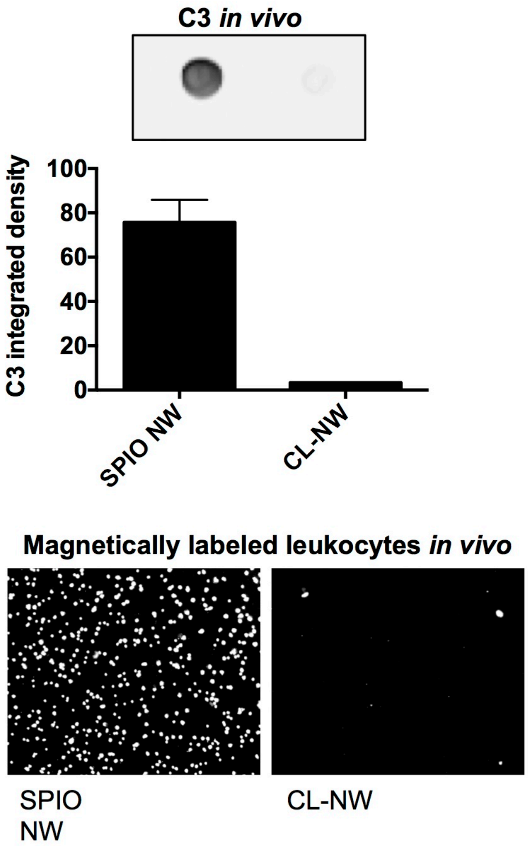

Supplemental Figure S3: C3 opsonization in vivo (upper panel and graph) and leukocyte uptake in vivo (lower panel) of SPIO NWs and CL-NWs. The experiment was performed as described in Fig. 6.

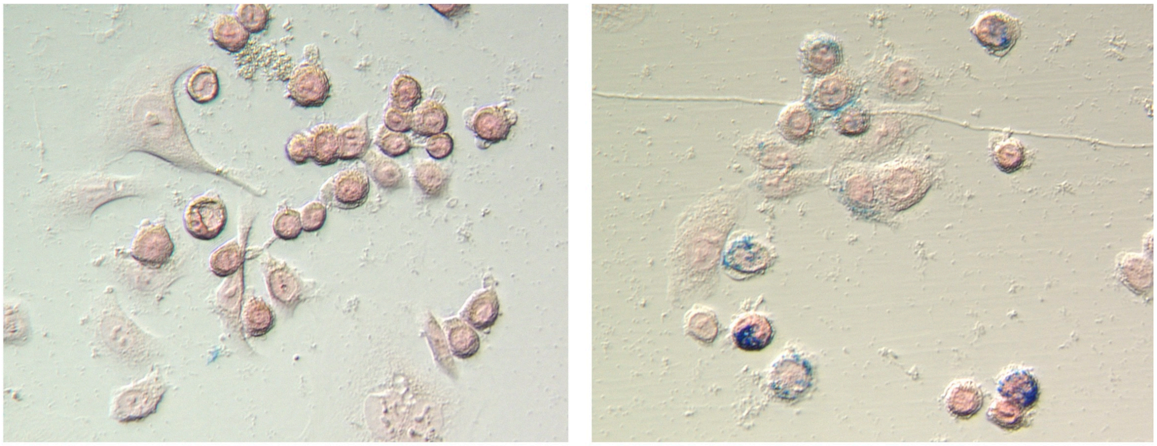

CL-NW-PEG

CL-NW-PEG-Ab (8)

Supplemental Figure S4: Uptake of NWs by SKBR-3 human breast cancer cells: The cells were incubated with non-targeted CL-NWs-PEG and trastuzumab-modified CL-NWs-PEG-Ab (8 IgG/particle) for 6 h and the internalized iron was detected using Prussian blue stain after cell fixation.
